# Balance between Retroviral Latency and Transcription: Based on HIV Model

**DOI:** 10.3390/pathogens10010016

**Published:** 2020-12-29

**Authors:** Aneta Pluta, Juan P. Jaworski, César N. Cortés-Rubio

**Affiliations:** 1 Department of Biochemistry, National Veterinary Research Institute, 24-100 Puławy, Poland; 2 Consejo Nacional de Investigaciones Científicas y Tecnológicas (CONICET), Instituto Nacional de Tecnología Agropecuaria (INTA), Instituto de Virología, Nicolás Repetto y De los Reseros (s/n), CP1686 Hurlingham, Buenos Aires, Argentina; jaworski.juan@inta.gob.ar; 3 Centre for Research in Infectious Diseases, National Institute of Respiratory Diseases, Tlalpan 4502, 14080 Mexico City, Mexico; cesar.cortes@cieni.org.mx

**Keywords:** retroviruses, human immunodeficiency virus type 1, HIV-1, terminal repeat region, LTR, regulation transcription, latency and CRISPR/dCas9

## Abstract

The representative of the *Lentivirus* genus is the human immunodeficiency virus type 1 (HIV-1), the causative agent of acquired immunodeficiency syndrome (AIDS). To date, there is no cure for AIDS because of the existence of the HIV-1 reservoir. HIV-1 infection can persist for decades despite effective antiretroviral therapy (ART), due to the persistence of infectious latent viruses in long-lived resting memory CD4+ T cells, macrophages, monocytes, microglial cells, and other cell types. However, the biology of HIV-1 latency remains incompletely understood. Retroviral long terminal repeat region (LTR) plays an indispensable role in controlling viral gene expression. Regulation of the transcription initiation plays a crucial role in establishing and maintaining a retrovirus latency. Whether and how retroviruses establish latency and reactivate remains unclear. In this article, we describe what is known about the regulation of LTR-driven transcription in HIV-1, that is, the cis-elements present in the LTR, the role of LTR transcription factor binding sites in LTR-driven transcription, the role of HIV-1-encoded transactivator protein, hormonal effects on virus transcription, impact of LTR variability on transcription, and epigenetic control of retrovirus LTR. Finally, we focus on a novel clustered regularly interspaced short palindromic repeats-associated protein 9 (CRISPR/dCas9)-based strategy for HIV-1 reservoir purging.

## 1. Introduction

The human immunodeficiency virus type 1 (HIV-1) belongs to the family of *Retroviridae*, subfamily *Orthoretrovirinae*, and genus *Lentivirus*. HIV-1 is firmly associated with the acquired immunodeficiency syndrome (AIDS) [1]. Highly pathogenic *lentiviruses*, after integration of double-stranded viral DNA into cellular genome, activate transcription of the viral genome. After synthesis of *viral* nucleic acid and formation of several viral proteins, to complete the viral life cycle, progeny virions are produced [2]. The efficiency of the initial transcription of integrated DNA from 5′ long terminal repeat (LTR) region promoter determines the level of viral RNA in an infected cell. Proviral 5′ LTR promoter contains numerous cis-regulatory elements, which modulate the rate of viral transcription initiation. However, certain cell types and the cell differentiation processes with respect to diversity of cell activation signals may contribute to substantial variations in transcriptional activity of LTR [3]. All these variables generate a remarkably broad range in HIV-1 gene expression level. Contrary to simple retroviruses (avian leukemia virus and murine leukemia virus), regulation of *lentivirus* gene expression involves both cellular and virally encoded regulatory factors. Consequently, RNA production in HIV-1 infection is highly variable. 

The latently infected cells are a source of viral reactivation and lead to marked increase of the viral load after a pause of highly active antiretroviral therapy (HAART). In this context, a better understanding of the molecular mechanisms responsible for the regulation of proviral latency and reactivation would define rational strategies aimed at purging the HIV-1 reservoirs in treated patients. The regulation of gene expression in HIV-1 is complex and requires multiple steps, including chromatin organization, allowance of transcription machinery, mRNA processing and its transport to the cytoplasm, translation and posttranslational processes. In this review, we describe those viral and cellular elements involved in regulation of HIV-1 transcription, that is, (1) LTR regulatory elements, (2) variety of enhancers, (3) activation of LTRs by virus-encoded Tat protein, (4) role of hormones in regulation of LTR transcription, (5) impact of naturally occurring functional mutations in LTRs, and (6) epigenetic control of retrovirus LTR. In addition, we discuss a novel clustered regularly interspaced short palindromic repeats-associated protein 9 (CRISPR/dCas9)-based strategy for HIV-1 reservoir purging.

## 2. LTR Regulatory Elements

Retroviruses integrate into host DNA as proviruses that are flanked by LTRs at each end of the viral DNA. Transcription of proviral DNA is catalyzed by cellular RNA polymerase II (RNAPII) and initiated at the U3 end of 5′ LTR. Each LTR is composed of three regions: unique 3′ (U3), repeated (R), and unique 5′ (U5). U3 occupies most of the LTR and plays an important role in the induction of retroviral transcription, since it contains the viral promoters and other cis-active elements required for the modulation of promoter activity. The TATA box, located within the LTR promoter element, provides the binding site for RNAPII, determining the site of initiation and also affecting the efficiency of the initiation of transcription [4]. 

The U3 region of HIV-1 LTR contains the crucial regulatory elements for the core promoter region: three specific protein 1 (Sp1) sites and TATA box; for the enhancer region: two nuclear factor-κB sites (NF-κB) and one nuclear factor of activated T-cells (NF-AT) site; for the modulatory region: three CCAAT/enhancer binding protein (C/EBP) sites, the activating transcription factor/cyclic AMP response element binding (ATF/CREB) region, two NF-AT sites, two activator protein *1* (AP-1) sites, one upstream stimulatory factor Ets/PU.1, and one T-cell specific transcription factor/lymphoid enhancer binding factor (TCF/LEF-1) [5,6,7,8].

In HIV-1, the following regulatory sequences downstream of the transcription start site are as follows: the initiator (Inr), the inducer of short transcripts (ITS), and trans-activation responsive element (TAR). TAR forms an RNA stem-loop structure, which recruits the virally encoded transactivator protein (Tat) to the LTR to modulate the activity of the viral promoter [1]. In addition, HIV-1 LTR consists of several substantial transcription factor (TF) binding sites including AP-1 sites, an AP-3-like (AP-3L) sequence, C/EBP/NFAT (nuclear factor for activated T cells) downstream binding site (DS3), two downstream sequence element (DSE) sites, one downstream binding factor (DBF-1) in R region, and two Sp1 binding sites and gag leader sequence (GLS) in the U5 [9]. Enhancer functions have been also mapped to the *gag*–*pol* regions of simian immunodeficiency virus (SIV) and HIV, but their role in the virus replication has yet to be established.

The transcription of *Lentiviruses* is regulated by the interactions between numerous and different viral proteins and transcription factors with binding sites located in the 5′ LTR. Most of regulatory elements encompass the U3 region. Regulatory elements situated in R and U5 regions may improve the promoter and enhancer strengths and provide a broad viral response for stimulating factors and control transcription in cell-type-dependent manner. 

## 3. A Variety of Enhancers with Regulatory Functions

The HIV-1 mainly infects CD4+ T cells, monocytes, and macrophages, and in a lower proportion also dendritic cells (DCs) and microglial cells [6]. HIV-1 enhancer sequence consists of two NF-ĸB binding sites and three adjacent Sp1 binding sites that are required for viral transcription [5]. Other factors shown to bind the enhancer include Ets, PU.1, NF-AT, C/EBP, AP-1, cAMP response element-binding protein/ activating transcription factor (CREB/ATF), upstream stimulatory factor (USF), Sp1, Sp3 and chicken ovalbumin upstream promoter transcription factor (COUP-TF) and they play role in enhancing the transcription [6] (Table 1). 

This variety of binding sites may result in maintenance reverse latency in some cells. As an example, NF-κB transcription factor binding to enhancer sites within LTR activate viral transcription in most HIV-1-infected types of cells [10]. The transcriptional activity of the NF-κB and other transcription factors in primary immune cells versus transformed cell lines is listed in Table 2. 

In activated CD4+ T lymphocytes, the Sp1 transcription factors are not sufficient to mediate transcription and further binding NF-ĸB and NF-AT cellular factors to the LTR enhancer region is required to activate transcription [6]. In addition, the USF, Ets, NF-IL-6 and CREB proteins facilitate efficient transcription [6]. In long-lived latently infected CD4+ T cells, NF-ĸB and NF-AT, as key factors for initiation of HIV-1 transcription in these cells, are present in very low nuclear concentrations. In addition, Cyclin T1 protein levels are also very low in comparison to activated T cells. For that reason, the above mechanisms have been proposed to be probably involved in CD4+ T cell latency [52]. 

In monocyte–macrophage lineage cells, regulation of HIV-1 transcription varies considerably during macrophage differentiation, as numerous transcription factors are expressed in a differentiation-dependent manner. In monocytes, LTR activity may be regulated during their differentiation stages by changes in the Sp1 (activator):Sp3 (repressor) ratio [30]. 

Increased permissiveness of macrophages for HIV-1 replication leads to expression of the cofactors utilized for Tat transactivation of the LTR, and this leads to a high level of HIV-1 transcription. There are numerous studies supporting that microglial cells are susceptible to HIV-1 infection and can be latently infected, constituting a major reservoir in the brain. In contrast to the monocytes, NF-κB, AP-1, and NFAT proteins are constitutively localized in the nucleus of microglial cells, and the Sp1 expression predominates over the Sp3 [6]. Interestingly, latently infected microglial cells can be reactivated by cytokine stimulation. In contrast to other reservoirs, the NF-kB and Sp1 binding sites are sufficient for HIV-1 transcription in microglial cells [53]. Contrary to CD4+ T cells, which express only Sp1, microglial cells produce both Sp1 and Sp3; the latter acting as transcriptional repressor. In addition, C/EBPɤ is expressed and acts as repressor by competing with the transcriptional activator C/EBP (Table 2) [54]. 

To conclude, the LTRs play a significant role in cell-type-specific expression of the proviral genome. HIV-1 enhancer sequences contain many binding sites providing mechanisms for a broad viral response to extracellular factors and regulate transcription in the cell-type-dependent manner. These observations thus emphasize the differences in mechanisms underlying HIV-1 latency between infected cells. 

## 4. Transactivation of LTR by Virus-Encoded Tat Protein

*Lentiviruses* are capable of promoting the rate of their gene expression through virus-encoded transactivator proteins. Activation occurs by binding of Tat HIV-1 protein to a specific sequence adjacent to 5′ trans-activation response (TAR) element RNA transcript [55,56,57]. Tat protein of HIV-1 (and related *Lentiviruses*) interacts with the viral RNA transcript, through a unique RNA regulatory segment of the LTR termed transactivation-responsive element (TAR). The TAR secondary RNA structure is formed from transcription of the +19–43 tract in the LTR R region [58]. Various mechanisms of HIV-1 Tat transactivation have been proposed. One model suggests overriding transcription terminations, since in the absence of Tat transcripts that initiate in LTR pause after synthesis of about 70 nucleotides [1]. It has also been proposed that in early steps of viral transcription, the complex of positive transcription elongation factor b (P-TEFb) composed of Cyclin T1 (CycT1) and cyclin-dependent kinase 9 (CDK9) is recruited to the LTR via nuclear factor kappa B (NF-κB). The recruitment of Tat and P-TEFb to the TAR hairpin facilitates phosphorylation of RNAP II, which increases their combined effectiveness and prevents premature termination [1,58,59]. On the other hand, several investigations revealed that NF-κB can promote both transcription initiation and elongation complex, at a similar level to that of Tat, in a manner independent of Tat. The NF-κB transcription factors induce LTR regulation via interaction with binding sites located within the enhancer region [59]. Deletion of the NF-κB binding sites strongly reduces basal, as well as Tat-transactivated, LTR activity. The Tat proteins activate NF-κB through a IκB kinase (IKK), which accelerates the degradation of IκB, a protein that regulates NF-κB activity by binding NF-κB and translocating to the nucleus [59,60]. In vitro model systems support an alternative hypothesis where Tat initiates transcription through a protein–protein interaction with the Sp1 transcription factor. This paradigm is supported by findings that nucleotide changes within the cis-acting elements recruiting Sp factors to the HIV-1 LTR reduce Tat-mediated LTR activity [60]. 

Additionally, viral protein R (Vpr) is another viral accessory protein capable of enhancing the activity of the HIV-1 LTR. Vpr can bind to histone acetyltransferases (HAT) CREB-binding protein and p300, glucocorticoid receptor, CycT1, and Tat to activate transcription [61,62]. Vpr can also activate NF-κB-directed transcription (reviewed in [1]). HIV-1 LTR C/EBP and NF-κB complex demonstrates a high affinity for Vpr and a low affinity for C/EBPβ during late-stage HIV in brain cells from patients with HIV-associated dementia (HAD) [61,62]. In addition, Kilareski and co-workers identified specific Tat variants derived from HAD brain, which were defective in LTR transactivation, however still were able to activate promoters of the other proinflammatory cytokine genes [30]. Collectively, in the tissues of the brain, Tat may become less transcriptionally competent, however, in this situation, Vpr may facilitate HIV-1 replication by enhancing transcription in the absence of a fully active Tat. On the other hand, Razooky and co-workers suggested that Tat can control a viral reservoir in infected resting and memory CD4+ T cells, even if the Tat level in these cells is low. They found that Tat mutants exaggerated lower levels of HIV-1 expression in the resting cells [63,64]. In addition, Chakraborty and co-workers data indicated that Tat promotes latency by generating a negative feedback loop at later stages of infection, which leads to the silencing of HIV-1 promoter [65]. 

The primary function attributed to Tat is the transactivation of HIV-1 promoter. Additionally, it has been demonstrated that Tat enhances HIV-1 virulence by interacting with various cellular proteins in order to induce T cell apoptosis, co-receptor regulation, and cytokine induction in the host cells [66,67,68]. The effect of Tat on many viral activities in the host cell contributes to the pathogenesis of HIV-1, pointing to this molecule as a potential target for HIV-1 therapy, for example, by blocking viral replication by targeting Tat [69,70,71,72]. The Tat naturally occurring polymorphisms are usually caused by viral mutational escape from CD8+ cytotoxic T lymphocyte (CTL) recognition. The host immune responses mediated by CTLs and less by CD4+ T lymphocytes and B lymphocytes may potentially force selective pressure towards Tat diversity and affect its activity [73,74]. It has been proposed that variations in Tat sequence could modulate transactivation and have implications on HIV-1 latency and the reactivation phase. Ronsard and co-workers reported that the Tat variants with a change of S46F were able to significantly enhance LTR transactivation compared with wild-type Tat [75]. Additionally, the change of S46F caused strong Tat interaction with TAR in in vitro and in silico models. In contrast, a naturally occurring change of the C22S in HIV-1 Oyi strain reduced Tat transactivation activity and was linked with long-term nonprogressive infections [76]. Furthermore, other naturally occurring polymorphisms within Tat identified in HIV-infected patients at acute and/or early infection phase (i.e., P10S, W11R, K19R, A42V, and Y47H) have been shown to significantly impair transactivation activity in the infected CD4+ T lymphocytes [77]. These data suggest that certain naturally occurring changes can change Tat transactivation activity.

The infected lymphocytes rapidly produce great numbers of viral particles, and it is clear that Tat protein triggers this process. Clones with nonsense changes are unable to replicate and thereby disappear from the spectra in vivo. However, as the infection progresses, some naturally occurring changes in Tat can change its immunogenic properties, prevent transactivation, and may influence viral latency. Nevertheless, it remains unclear to what extent CTL escape changes occurring in the Tat epitope may affect the HIV-1 latency kinetic from establishment to reversal stages [78].

## 5. Regulation of LTR Transcription through Hormonal Signaling

HIV-1 LTR includes regulatory elements that can be modulated by different hormonal transactivating factors. For example, the estrogen receptor-1 (ESR-1) suppresses HIV-1 transcription and is required to maintain HIV-1 latency in cells harvested from HIV-positive patients and cultivated in vitro [79]. It has been demonstrated that ESR-1 associates with β-catenin, forming a corepressor complex for the HIV-1 LTR. Although it has been shown that ESR-1 accumulates at the LTR in the presence of β-estradiol, it is unclear if it directly binds to the LTR or if it exerts an indirect modulatory effect. In this regard, Sp1, which binds to three regions of HIV-1 LTR (143 nt from the transcription initiation site), has been proposed as a potential mediator of ESR-1 recruitment on the LTR [80,81]. Altogether, these data suggest that estradiol could potentially repress HIV-1 replication by altering HIV-1 transcriptional activation. In this regard, a negative effect on HIV-1 replication was observed when HIV-infected peripheral blood mononuclear cells (PBMCs) were cultured in vitro with the addition of either 17-β-estradiol (E_2_) or progesterone (P_4_) [82]. 

Thyroid hormone (T3) receptor alpha (T3Rα) is another host hormonal element that modulates HIV-1 LTR transcription [83]. This regulation is mediated by the thyroid hormone response element (T3RE) located at −74 to −50 nt from the transcription initiation site of HIV-1 LTR and coinciding with the Sp1 docking element [84]. Interestingly, in the absence of T3, T3Rα binds to the LTR, leading to a local reduction of histone acetylation. Consequently, T3Rα represses the HIV-1 promoter in a histone deacetylase-dependent manner. In contrast, when it is bound to T3, T3Rα leads to chromatin remodeling, including histone acetylation and chromatin disruption, which is essential in the activation of LTR-derived transcription by T3Rα [85]. 

Two glucocorticoid response elements (GRE) have been described for HIV-1; the first located within the LTR and a second within the Vif open reading frame. Recombinant glucocorticoid receptors were found to interact with GRE located within -259/-264 region of the LTR [86,87]. Furthermore, an increase in HIV-1 replication was observed in lymphoid and monocytoid cell lines infected with HIV-1, and treated with dexamethasone (a synthetic glucocorticoid). In addition, it has been demonstrated that by interacting with the GRE, progesterone could augment HIV-1 replication [88]. In a separate study, Mitra and co-workers showed that dexamethasone increased HIV-1-directed gene expression in monocytoid cell lines; however, the same treatment suppressed HIV-1 expression in lymphocytic B and T cell lines [89]. These results suggest that the glucocorticoid receptor binding site in HIV-1 LTR could mediate either activation or repression of viral gene expression depending on the target cell type. 

## 6. Impact of Naturally Occurring Functional Mutations in LTRs

High variability is a feature of retroviruses as it is for most other RNA viruses. The genetic variability observed for retroviruses is a consequence of the high error rate of its reverse transcriptase (RT), which lacks a proofreading activity. Natural occurring and selected mutation within the proviral sequence has important implications for virus replication, immune escape, and disease progression. Escape mutations can be easily understood if we consider HIV-1 infection and how the variability of envelope protein (Env) contributes towards immune evasion within a single HIV-1-infected individual. Moreover, considering the clinical outcome associated with persistent immune evasion by HIV-1, fixed mutations, incorporated by RT during provirus-DNA synthesis, can be located within the whole genome, including the LTRs. Particular variations within the HIV-1 LTR sequence impact viral replication and expression patterns amongst different HIV-1 subtypes. In this regard, increased LTR function has been associated with a higher copy number of cis-acting transcription factor binding sites [90,91]. It has been shown that a natural HIV-1 variant with four Sp1 sites in the LTR had a stronger promoter activity and viral replication rate than the typical LTRs with only three Sp1 sites [90]. The other example may be the natural HIV-1 variant from subtype C containing three NF-κB binding sites in LTR. These clade C variants had higher activity in activating viral gene expression than LTRs from HIV-1 B subtypes with the conventional two NF-κB binding sites. Conversely, HIV-1 variant CRF01_AE included a single copy of NF-κB binding site, since the second NF-κB site, usually observed in other subtypes, was replaced by GA-binding protein (GABP) binding site [92,93]. This conversion reduced the response upon host factors stimulation and led to latent infection. In support of this, a single NF-κB binding site was observed in less pathogenic *lentiviruses* with a relatively low replication rate [94]. Other examples of sequence variation in the LTRs of different retroviruses are presented in Table 1. It has been demonstrated that single point mutations within the LTR can affect retrovirus host cell tropism and pathogenesis. For instance, a single point mutation within the TATA box of HIV-1 variant CRF01_AE (TATAA > TAAAA) reduced transcription activity, by interfering with the efficiency of assembly of the TBP–TFIIB–TATA complex and consequently, decreasing the recruitment of RNA polymerase II [92,95,96]. Another group showed that a C to T substitution at the Sp1 site III of HIV-1 LTR was associated with more severe disease progression in HIV-infected individuals [97]. 

Similarly to the other retroviruses, HIV-1 shows a high degree of genetic variation. The error rate in bovine leukemia virus (BLV) is 4.8 × 10^−6^ nucleotides, as compared to 2.5–5.9 × 10^−4^ for purified HIV-1 RT and 3.4 × 10^−5^ measured during single-cycle HIV-1 infection, 5.9 × 10^−5^ for avian myeloblastosis RT, 3.3 × 10^−5^ for Moloney murine leukemia virus RT, and 1.2 × 10^−5^ nucleotides for avian spleen necrosis virus RT [98,99,100,101]. 

A morphological and functional variety of cells from different tissue types stimulates *lentivirus* genome to evolve in a tissue-compartmentalized manner. Tissue compartmentalization of human immunodeficiency viruses and animal *lentiviruses* is well documented [102,103,104,105,106]. HIV-1 compartmentalization has been observed in multiple tissues and anatomic compartments, including spleen, lymph node, breast, gut, genital tract, lung, liver, kidney, and brain. The presence of tissue-adapted variants plays an important role in the pathogenesis of HIV-1 disease. In addition to the differences extending to Env, Nef, and Tat, compartmentalization is associated with nucleotide polymorphisms within the LTR that result in modifications of the transcription factor binding sites and alterations in the efficiency of the sites to bind proteins that play an important role in regulating viral gene expression [107,108,109]. Microglial cells, the central nervous system (CNS) resident macrophages, present several differences. In contrast to other tissue compartments, microglial cells are characterized by permissiveness to HIV-1 infection, reduced immune surveillance, resistance to apoptosis and cytopathic effects, reduced antiretroviral drug efficiency, and the unique CNS cellular microenvironment. Therefore, they are considered as a reservoir of latent HIV-1. 

In a series of studies led by Dr. Brian Wigdahl, the authors described an association between the C/EBP binding sequence variability, tissue tropism, and disease progression of HIV-1. They showed that thymidine to guanosine (T > G) substitution at position 6 of the C/EBP site I resulted in increased C/EBP binding and LTR activity, and observed that the frequency of such particular mutation was higher in CNS-derived viruses compared with those viruses obtained from peripheral blood [53]. In a separate study, the authors observed that C/EBP site II was conserved. Moreover, the presence of a high-affinity site within C/EBP site II in CNS-derived LTRs was associated with levels of viral replication and development of HIV-1-associated dementia (HAD) [110]. Conversely, low-affinity C/EBP sites were found preferentially in regions of the brain characterized by low rates of viral replication [53]. Overall, these studies highlight that naturally occurring mutations at C/EBP sites might impact HIV-1 pathogenesis, as well as the maintenance of latent reservoirs in the CNS. 

In addition, several other mutations within and/or surrounding the binding sites for Ets-1, USF, C/EBP, AP-1, and Sp1 core promoter region were linked to a common phenotype (i.e., transcriptional activity) particular to LTRs of proviruses obtained from CNS [111]. Although the molecular mechanisms remain to be elucidated, it was reported that CNS LTRs had lower basal transcriptional activity compared with non-CNS LTRs; however, they retained their ability to be activated by HIV-1 Tat [111,112]. 

In a separate study, the authors described a high degree of heterogeneity in Sp3 site from both CNS and lymphoid compartment-derived isolates, however, polymorphisms in adjacent sequences were found at a higher frequency in the CNS-derived isolates [113]. The authors concluded that alterations in sequences flanking the Sp-binding motif of brain-derived HIV-1 strains may reflect functional differences when compared to the lymphoid-derived viruses [113]. 

## 7. Epigenetic Control of Retrovirus LTR 

### 7.1. Histone Acetylation and Deacetylation 

HIV-1 proviral DNA transcription depends on histone post-translational modifications, mainly acetylation and methylation status [114,115]. Major histone acetyltransferases (HATs) are recruited towards the HIV-1 proviral DNA by transcription factors NF-κB, NFAT, and C/EBPβ and modify key lysines on H3 and H4 on the 5′-LTR region [10,116,117,118,119]. Histone acetylation modifies the structure and accessibility of DNA for transcriptional activators, initiation factors, and RNA polymerase II (RNAPII), therefore it is associated with transcriptional stimulation. 

Conversely, the Yin Yang protein 1 (YY-1), NF-κB (p50–p50 homodimer), and C- promoter binding factor (CBF-1) allow recruitment of histone deacetylases (HDACs), which repress HIV-1 transcription and promote the establishment of HIV-1 latency [120,121,122]. Histone deacetylation may be important for blocking HIV-1 gene expression in resting CD4+ T lymphocytes. Additionally, retinoblastoma binding protein 4 (RBBP4) and COUP-TF1 may play an important role in inhibiting HIV-1 transcription by recruiting the chromatin remodeling complexes. Briefly, RBBP4 binds to the HIV-1 LTR and recruits COUP-TF1 and HDAC1/2 to the HIV-1 LTR, which further modulate local H3 deacetylation [123]. More studies focusing on HDAC inhibitors and their role in stimulating the retrovirus release from latently infected CD4+ T cells are warranted since they might have clinical utility by reducing the latent reservoir [124]. 

### 7.2. Histone Methylation

In addition to histone lysine acetylation (Lys-Ac), the histone lysine methylation (Lys-Me) may play dual roles in the regulation of HIV-1 transcription. The methylation of H3 at Lys9 (H3K9me) by histone methyltransferase (HMT) is associated with inactive genes and constitutive heterochromatin [125]. Additionally, in mammalian cells, different patterns of H3K9 methylation can occur, that is, suppressor of variegation 3-9 homologue 2 (Suv39H2), Suv39H1, and histone-lysine N-methyltransferase (SETDB1)/ERG-associated protein (that add the mark H3K9me3), G9a, (that add the mark H3K9me2), and G9a-like protein (GLP)/EuHMTase1 (that add the marks H3K9me1 and H3K9me2), highlights the importance of silencing through histone modification mediated by HIV-1 [115,125,126,127]. Since H3K9 methylation plays an essential role in chromatin-mediated transcriptional silencing, the biology and molecular mechanisms of H3K9 methylation generated by different HMTs and their association with HIV-1 gene repression need to be further studied. 

Jiang and co-workers showed that the proviral reservoirs (particularly intact proviral sequences) of elite controllers (ECs) had the following characteristics: (1) consisted of oligoclonal to near-monoclonal clusters, (2) were integrated at highly distinct sites, (3) the sequences were integrated in centromeric satellite DNA and in Krüppel-associated box domain (associated with heterochromatin features), (4) the integration sites were enriched in H3K9me3 (chromosomes 7 and 19), and (5) the integration sites were in chromosomal regions susceptible to DNA methylation [128]. On the basis of these considerations, Jiang and co-workers hypothesized that ECs are maintained over time in intact proviral sequences in a deep and long-lasting latency state, possibly due to integration into regions that are not permissive to active HIV-1 transcription [128]. It is probable that distinct reservoir configuration is not related to the integration site location, but instead can be the result of immune selection forces that preferentially eliminate proviral sequences that are more permissive to virus transcription. Remarkably, this work demonstrated for the first time that one EC (ECL2/Loreen Willenberg) could achieved a sterilizing cure of HIV-1 infection without medical intervention, since the authors were unable to detect intact proviral sequences despite wide analyses of more than 1.5 billion PBMCs (it is estimated that EC represent <1% of the HIV+ patient population) [129]. 

Many viral promoters can be targeted for silencing through conversion to facultative heterochromatin. The facultative heterochromatin is created by the polycomb repressor complex 2 (PCR2), responsible for methylation of the Lys 27 (di and tri) on H3 (H3K27me2/3) [130]. Friedman and co-workers made chromatin immunoprecipitation experiments using latently infected Jurkat T cell lines and demonstrated the high levels of enhancer of zeste homolog 2 (EZH2) at LTR from silenced HIV-1 proviruses rapidly decreased following proviral reactivation. It was suggested that PRC2-mediated silencing is required in HIV-1 latency [130]. To conclude, the inhibitors of Suv39H1/2, SETDB1, G9a, (GLP)/EuHMTase1, and EZH2 may be potential therapeutic targets useful in reactivation strategies to eradicate latent HIV-1. While in ECs they are near to achieving a functional cure, the sterilizing cure is more distant. In the general HIV-1 + population, the picture is more complicated. We could still aspire to achieve a functional cure using the knowledge acquired in epigenomics (silencing) in the near future, however, the sterilizing cure could be achieved in a more distant future, using the epigenomic editing tools (which are reviewed later in the text). 

### 7.3. ATP-Dependent Chromatin Remodeling Complexes

The ATP-dependent chromatin remodeling complexes are potent molecular motors, which use energy from ATP hydrolysis to lose the DNA-histone contact and change the accessibility of DNA [131,132]. The members of the family of remodeling complexes called SWItch/sucrose non-fermentable (SWI/SNF) contain either BRM or BRG1 proteins as catalytic subunit (ATPase) [131,132,133,134,135,136]. The SWI/SNF family includes brahma-related gene-associated factor (BAF) and polybromo and brahma-related gene 1 (BRG1)-associated factor (PBAF). The BAF and PBAF complexes are remodeling the nucleosomes, located near the transcription start site.

The BAF complex contains BRG1 or BRM (ATPase) and specific subunits BAF250a/b [137,138], while the PBAF complex contains BRG1, or no BRM (ATPase), and subunits BAF180 and BAF200, SAYP, and BRD7 [139,140,141,142,143]. It is important to note that the type of subunits that make up the complex determine if the complex will act as an activator or repressor of transcription. For example, in chronically HIV-1-infected cells, Duyne and co-workers found that BAF53-containing complexes are involved in suppression of HIV-1 transcription [144]. On the other hand, BAF53 has been found in both cores (BAF and PBAF). The BAF and PBAF complexes play diverse roles in HIV-1 transcription.

In 2011, Rafati and co-workers revealed that the activation of a suppressed HIV-1 promoter in response to the loss of BAF250a suggests that the BAF complex is required for LTR repression and is necessary to keep HIV-1 latent [145]. The experimental data suggested that the BAF complex is recruited early to the HIV-1 LTR independently of Tat. However, the activation of the LTR is Tat-dependent and is carried out by the PBAF complex [145,146].

The experimental results have allowed a model of the regulation of transcription given by BAF and PBAF to be built. BAF complex binds to the LTR and, by use of the energy of ATP, is able to position nuc-1 from the DNAaseI-hypersensitive site (DHS1) (leaving free the transcription factors binding sites) until an energetically less favorable sequence (with lower affinity) found immediately downstream of the transcription start site (TSS); in this way, BAF complex is able to represses the activity of the LTR. During transcriptional silencing, the Tat protein is methylated (Tat-met) by HMT called SETDB1 (in Lys 50 and 51), and Tat-me can interact with DNA methyltransferase 3A (DNMT3A) and Histone deacetylase 1 (HDAC1) to form an inhibition complex and create heterochromatin [145,147]. The activation occurs when the BAF complex dissociates from the LTR and Tat protein is recruited to the HAT p300, which carries out the acetylation of histones and Tat and where the PBAF complex is finally recruited to the LTR by Tat-ac [145]. Easley and co-workers showed that acetylated Tat (86 or 101) interacts efficiently with BAF200 (a component of PBAF) [146]. PBAF is able to activate LTR transcription when repositioning the nucleosome nuc-1 away from the transcription start site, moving it downstream until it reaches a sequence with lower affinity [145,148]. Finally, the BRG1 enzyme, integrase interactor 1 (INI-1), BAF250, and SETDB1 are considered therapeutic targets to be inhibited in order to activate the HIV-1 reservoir.

### 7.4. DNA Methylation

Chromatin modifications are linked with direct changes in DNA (i.e., methylation of the cytosine C-5 position in the promoter region of HIV-1 associated to viral latency). DNA methylation leads to changes in the affinity of DNA for transcription factors, structural changes in DNA, displacement of specific nucleosomes, and interaction of histone H1 to DNA [149]. There is a negative correlation between the degree of DNA methylation and transcriptional activity. Therefore, regions with a high degree of DNA methylation correspond with transcriptionally inactive regions [149]. Once HIV-1 genome (DNA) is integrated into the host genome as a provirus, it can be subject to DNA methylation as it becomes part of the host cell genome. DNA methylation occurs in cytosine residues in a CpG context. For that reason, the HIV-1 5′-LTR methylation in a CpG context results in the silencing of the proviral genome [150,151]. Although 5′ and 3′ HIV-1 LTRs have high identity percentage (97.6% in reference sequence HxB2), Klaver and co-workers showed that (1) the 5′ and 3′ LTRs of an HIV-1 provirus can be transcriptionally active, (2) the absolute levels of transcription were much higher for the 5′-HIV-1 LTR than for the 3′-HIV-1 LTR, (3) the 5′-LTR is the major transcriptional promoter of an integrated HIV-1 provirus [152]; only the 5′-HIV-1 LTR can generate transcripts with a size near that of a complete genome, while the 3′-HIV-1 LTR can generate transcripts that include the R-U5 integration site, and (4) the transcriptionally active 5′-LTR suppresses 3′-LTR function. It is possible that usage of 5′-LTR could induce a transcriptionally unfavorable chromatin topology on the 3′-LTR promoter element (cis-acting, epigenetic suppression model). 

Supporting this idea, it is known that the DNA methylation status in the 5′ and 3′ HIV-1 LTRs may be different. Ishida and co-workers showed that in the DNA from unstimulated human T cell lines latently infected with HIV-1 _LAV_ strain (ACH-2), among ten clones obtained from the 5′-LTR, six showed methylation of nine CpG sites, two clones had two unmethylated CpG sites and seven methylated CpG sites, and the two remaining clones had the nine hypomethylated CpG sites. In contrast, all ten clones from the 3′-LTR had the nine hypomethylated CpG sites. In this context, the 3′-HIV-1 LTR would become progressively more active as the 5′-HIV-1 LTR was repressed. This will have important implications beyond the integration site (neighbor gene) [124]. The HIV-1 LTR methylation in monocyte–macrophage lineage cells results in the HIV-1 transcriptional silencing of promoter, which leads to poor accessibility of transcription factors to their target sites on DNA. In chronically infected T cell lines and in latently infected lymphocytes in vivo, CpG sites in the 5′-LTR are found selectively hypermethylated [124,153]. The reduction of viral gene expression in these cases is explained by the interaction of methyl-CpG-binding proteins with methylated Sp1 binding sites in HIV-1 LTR, provoking an allosteric regulation of Sp1 transcription factors [154]. Furthermore, the transcription factors NF-κB and USF can also lose affinity for their methylated transcription factor binding sites in Jurkat cells stimulated by tumor necrosis factor α (TNFα) and phorbol 12-myristate 13-acetate (PMA) [30,155]. Interestingly, DNA methylation can be reversed by cytokines such as TNF-α, which leads to demethylation of the 5′-LTR and stimulation of the viral gene expression [124,156]. There are divergent results regarding the role of methylation of the HIV-1 5′-LTR promoter region on provirus expression in patients on antiretroviral therapy (ART). Blazkova and co-workers suggested that resting memory CD4 + T cells of individuals on long-term ART (median 11.5 years) possessed hypermethylated HIV-1 5′-LTR, while viremic individuals frequently had hypomethylated HIV-1 5′-LTR [157]. Nevertheless, they found also that in resting CD4 + T cells from aviremic individuals on short-term ART (median 2.9 years), the 5′-LTR methylation was rare [158]. Later, Trejbalova and co-workers found low levels of 5′-LTR methylation in resting CD4 + T cells from individuals on short-term ART (median: 2.3 years), as opposed to the individuals on long-term ART (median: 12.5 years), who presented high levels of 5′-LTR methylation. It was suggested that the stimulation of cells carrying latent proviruses may contribute to 5′-LTR methylation [159]. In a different study, Cortés-Rubio and co-workers revealed that HIV-1 5′-LTR methylation can be different over time in proviruses from circulating CD4+ T cells and is usually observed in aviremic individuals on short-term ART (from 30 to 54 months). In addition, they showed strong evidence that different LTR-methylation patterns can be associated with the combination of baseline and follow-up clinical features. Among the baseline characteristics are age, gain of CD4+ T cells at 48 months on ART, decrease in CD8+ T cells at 48 months on ART, the number of changes in the ART regimen, the time elapsed to achieve a undetectable plasma viral load (pVL), the third drug class in the ART regimen, and the nadir of CD4+ T cell count. Characteristics of the follow-up include a significant decrease in the proviral load during the follow-up, time on ART, and changes in proportions of circulating T cell subpopulations. Finally, the exploratory factor analysis using principal components (EFAPC) showed that the variables percentage of T stem cell memory (%T_SCM_), percentage of new CD4+ T cells (%T_NEW_), proviral load, and CpG methylation index had a high unique variance (>0.5 in each variable), highlighting that these four individual variables are relevant [160]. To conclude, DNA methylation suppresses the HIV-1 promoter activity, and the CpG methylation may play a relevant role in viral latency in vivo. Greater understanding of methylation mechanisms and their effect on viral latency seems valuable to guide the development of more specific and powerful HIV-1 therapies. 

On the other hand, the U3 region of HIV-1 LTR has few CpG sites (10 CpG sites in LTR derived from HXB2 strain), while there are DNAse hypersensitive sites, which explains the negative correlation between CpG methylation on HIV-1 5′-LTR and residual viral load in several studies. In addition, HIV-1 provirus is frequently integrated into transcriptionally active regions of the genome, which encode different T-lymphocytes genes [161,162]. Therefore, the transcriptional suppression of HIV-1 is not always associated with DNA methylation of 5′-LTR [155]. There are factors that could explain the contrasting results regarding the role of 5′-LTR methylation on HIV-1 latency. The most important factors include infection time, ART regimen, time on ART, cell type, CD4+ T cell subpopulation, rate of replacement of latently infected CD4 + T cells, proportion of defective proviruses, HIV-1 integration sites [158,159,163], and possibly a diet enriched in methyl group donors (e.g., biotin, folate, methionine, choline, and betaine). Importantly, it was recently suggested that several methodological factors could bias the conclusions on DNA methylation analyses, including the use of cell lines versus primary CD4 + T cells in vitro, frozen samples versus fresh samples, the type of bisulfite treatment, sampling bias, PCR amplification strategy, and sequencing technique [164]. Since DNA methylation could be a late event that reinforces the silencing of proviruses that were previously silenced by other epigenetic processes, the cell type and infection time are variables to consider in future DNA methylation analyses. In this regard, the chromatin modifiers and the biochemical mechanisms involved in the repression of the HIV-1 promoter (e.g., H3 deacetylation, H3K9 and H3K27 methylation, and DNA methylation) require further study. Interestingly, it has been shown that HDACi, currently used to eliminate HIV-1 latently infected cells as part of shock and kill strategies, could increase the level of DNA methylation of the HIV-1 5′-LTR and thus enhance the stability of the reservoir [159]. It is necessary to know the interrelationships amongst HDACi, DNMTs, and/or HMTs to efficiently reactivating a latent provirus.

## 8. CRISPR/dCas9 Based Strategy for HIV-1 Reservoir Purging

As we described in previous sections, most transcriptional repression mechanisms used by HIV-1 consist of cellular processes that are not specific to HIV-1 [165]. Consequently, the latency reversal agents (LRAs), used as a first step in shock-and-kill strategies, are nonselective drugs that act at a global genome level and not at specific loci. The lack of specificity of LRAs is the major obstacle to a safe and effective use of this particular anti-HIV-1 treatment.

In 2012, Jinek and co-workers published a work describing a family of endonucleases that use dual RNAs for sequence-specific DNA cut, allowing RNA-programmable genome editing [166]. The system is known as clustered regularly interspaced short palindromic repeats-associated protein 9 (CRISPR/Cas9). While CRISPR target any 20 bp genomic DNA sequence followed by the protospacer adjacent motif (PAM) 5′-NGG-3′ sequence [167], the Cas9 nuclease cuts 3 bp upstream from the PAM sequence within the complementary sequence to specific guide RNA (sgRNA) in eukaryotic cells. The high specificity and efficiency of the CRISPR/Cas9 system has allowed its application in many different biomedical areas, including anti-HIV-1 [168]. In this regard, CRISPR/Cas9 has been employed to remove HIV-1 DNA through gRNAs that target the LTR and/or essential viral genes [169]. It was first reported that Cas9- and LTR-targeting sgRNA inhibited the HIV-1 LTR-driven reporter gene expression [169]. Pinto and co-workers showed that the use of a Tat mimetic peptide on a model of HIV-1 latently infected cell lines and primary cells (PBMCs) HIV-1 infected in vitro improved the efficiency of HIV-1 editing by CRISPR/Cas9. They suggested that suppression of transcription could facilitate genetic editing [170]. 

The subsequent sequence analysis showed that CRISPR/Cas9 caused insertions and deletions in the LTR [169]. When the Cas9 nuclease cuts dsDNA, error-prone cellular repair mechanisms fix damaged DNA using two different mechanisms: (i) the first named classical nonhomologous end joining (NHEJ), which sticks together both ends of DNA, and (ii) the second mechanism named microhomology-mediated end joining (MMEJ), which requires alignment of internal microhomologous sequences to the site of DNA damage prior to repair [171,172]. Both mechanisms account for a chance of introducing mutations (insertions or deletions) during the repair process. HIV-1 genome integrates preferably into transcriptionally active regions of the host genome. During the chronic HIV-1 infection stage, the integration sites are mainly localized in loci associated with cell proliferation, differentiation, and/or oncogenesis processes [173,174,175,176]. For that reason, the use of the CRISPR/Cas9 editing system for the removal of HIV-1 proviral genome as a therapeutic approach could accelerate the appearance of cancer in treated individuals. 

Alternatively, it is possible to mutate the endonuclease activity domain from Cas9 (RuvC1 and HNH) to generate a “deactivated” Cas9 (dCas9). Although mutated dCas9 lacks endonuclease activity, it keeps binding capacity to sgRNA and DNA targets [166]. Compared to Cas9, dCas9 does not damage DNA [165], and for that reason, CRISPR/dCas9 system grants a safer and highly specific epigenetic editing tool for gene therapy [177]. This system consists of two indispensable parts: a DNA-binding targeting domain and a functional/effector domain. The functional domain/epigenetic effector (e.g., HAT, or a TET family enzyme) is fused to dCas9(i). In order to edit a specific region of DNA it is necessary to design an sgRNA that will direct dCas9 or Cas9 activity to a unique ~20 bp region of the genome. In order to target HIV-1, the different proviral genomes that constitute the viral reservoir within a single HIV-infected patient must be identified. A main challenge in designing sgRNAs to target the reservoir is to account for the huge HIV-1 variability characteristic of HIV-1. A single sgRNA is not capable of targeting all quasispecies present in a given patient. It has been hypothesized that such variability could lead to scenarios where a personalized set of sgRNA will need to be designed for each infected patient. Initial reports focusing on disrupting the highly conserved HIV-1-LTR sequences pointed out that up to 10 customized sgRNAs would be necessary to efficiently cover all proviral sequences present in the blood of patients under suppressive ART. Importantly, variability of the HIV-1-LTR sequences tends to decrease in patients under prolonged and effective ART, which could reduce the number of sgRNAs needed for the total removal of HIV-1 reservoir in patients with effective virus suppression [178]. Based on this, a more generalized approach for sgRNA design has been proposed. Using NGS data from the HIV-1 reservoir of 23 HIV-1-infected patients under suppressive ART, Dampier and co-wokers have proposed targeting any HIV-1 reservoir with a set of four consensus sgRNA [179]. According to their calculations, using an in silico algorithm, such a set should disrupt over 90% of proviral sequences with 90% efficacy. Finally, the functional domain/epigenetic effector (e.g., HAT, or a TET family enzyme) that is fused to dCas9 will perform the desired change at the specific site (determined by the sgRNA specificity) [180]. This CRISPR/dCas9-epigenetic effector tool can be used to change different epigenetics marks such as the following: 

1. The dCas9-p300 system, in which dCas9 is fused to the core domain of the human p300 HAT [181]. p300 HAT catalyzes histone acetylation at the specific site, determined by the union of the sgRNA, and when this modification is directed towards a promoter or enhancer, the transcription is activated [165]. Indeed, the dCas-p300 system was able to promote HIV-1 transcription in J-Lat cells [182]. 

2. The dCas9-TET system. DNA demethylation in mammals involves ten eleven traslocation enzyme (TET) that catalyzes the oxidation of 5-methylcytosine (5-mC), and that modified base can be removed through the base excision repair system (BER). The dCas9-TET1 fusion protein was capable of reactivating the GFP reporter gene fused to the Snrpn promoter, by inducing a 90% decrease in the DNA methylation levels in this promoter [183]. However, it is not clear whether the oxidized derivatives of 5-mC generated by TET (for example 5hmC, 5fC and 5caC) are intermediate molecules in DNA demethylation or are others epigenetic marks. It is important to note that the edit by use of TET enzyme requires the presence of the thymine DNA glycosylase (TDG) enzyme [184].

3. The dCas9- repressor of silencing 1(ROS1) system. Unlike animals, plants have enzymes that directly remove 5-mC without generating intermediate molecules. Devesa and co-workers fused the dCas9 protein with the catalytic domain of ROS1 (5-mC DNA glycosylase) from *Arabidopsis* and evaluated the reactivation of silenced genes by methylation in comparison to other dCas9-effectors [185]. They found that dCas9-ROS1, but not dCas9-TET1, was able to reactivate transcription of silenced genes by methylation through partial demethylation in many CpG sites of the specific sequence. The catalytic activity of dCas9-ROS1 was affected by the density of methylation in the target region, and its range of action was limited to the first 50 bp with respect to the sg RNA binding site. This study showed that reactivation induced by dCas9-ROS1 needed catalytic activity of DNA glycosylase/lyase, suggesting that 5-mC by cytosine edit was mediated by the BER system.

Some challenges related to the use of CRISPR/Cas9 and CRISPR/dCas9-epigenetic effector tool in HIV-1 therapy have been addressed, while others require future work. In order to grant safety and specificity of the treatment, off-target effects need to be excluded [186]. Avoidance of interaction with unspecific sequences (off targets) within the human genome can be achieved by a careful selection of sgRNAs in silico. Alternatively, the use of CRISPR/dCas9-epigenetic effector (vs. CRISPR–Cas9) would minimize the number of off-site mutations. If the target region is highly variable, the design of several sgRNAs will be necessary [178,179]. Since the HIV-1 reservoir remains poorly characterized, one challenge that requires further investigation is the development of a vector, carrying CRISPR/dCas9 and capable of targeting HIV-1 reservoir cells, specifically [187]. Securing an optimal delivery to all cells accounting for the HIV-1 reservoir in vivo remains a major, but not the only, limitation [187,188,189]. Other challenges include the control of the CRISPR/dCas9 system using an inducible promoter [190,191,192,193], the avoidance of the immune responses against the vector and exogenous proteins that constitute the system (e.g., Cas9 protein, of bacterial origin, and ROS1 protein of plant origin) [194], and to determine the complexity in editing: between transcriptionally active regions vs. transcriptionally inactive regions, and between subpopulations of CD4+ T Lymphocytes. Furthermore, clinical implementation of the CRISPR-based system will require an exhaustive evaluation of its performance in vivo, including the development of adverse effects, as well as pharmacologic and unintended side effects [165]. Several animal models for the study of HIV-1 (i.e., humanized-mouse and non-human primates) could be used to test the efficacy of the CRISPR/dCas9 system in vivo, before translating to clinical settings. In addition, they could be used to test the effect of the CRISPR/dCas9 system in particular body compartments (i.e., privileged sites for HIV-1, such as brain cells) [169] and to determine the combined effect of suppressive TAR combined with CRISPR/Cas9 or CRISPR/dCas9-epigenetic effector system [187], with the aim of eradicating the HIV-1 reservoir and allowing the functional and sterilizing cure of HIV-1.

## 9. Summary

HIV-1 infection persists despite long-term virus suppression following highly effective ART, due to the persistence of a latent HIV-1 reservoir. Understanding the molecular mechanisms underlying proviral genome expression and targeting HIV-1 latency mechanisms will be fundamental to achieve complete viral eradication. Although different posttranscriptional mechanisms participate in maintaining the latent state, it is usually a transcriptional blockade that contributes to the establishment of latency. 

**Figure 1:**
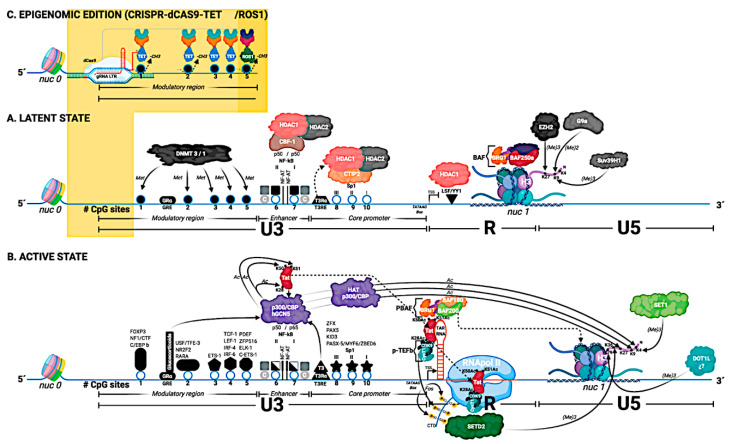
The structure of the HIV-1 5′ LTR region (based on the HXB2 reference sequence), and its genetic, hormonal, and epigenetic control are detailed in this figure. The 5′ LTR is divided into 3 regions: U3, R, and U5. The U3 region is divided into three functional domains: modulator region, enhancer, and the promoter core. The U3 region contains 10 CpG sites (represented by circles and numbered 1–10). The transcription factors that bind to the U3 region are located and represented by their name. In general, the transcription factor binding sites coincide with the CpG sites. (**A**) Latent State: nucleosome 0 (nuc 0) is located at the beginning of the U3 region of the 5′ LTR, and nucleosome 1 (nuc 1) is located in the R region of the 5′ LTR. In the U3 region of the 5′ LTR, transcription factors are recruited, in those binding sites that are accessible; in this Figure, NF-Kb binding site (homodimer p50/p50). The BAF complex (BAF 250a) is responsible for positioning nuc-1, downstream of the transcription start site (TSS), specifically in the R region. This image shows the elements that prevent both the initiation process of transcription (the binding of the transcription factor YY1) as well as the elongation of the transcript (nuc 1 in R region). Histone H3 from nuc 1 gradually acquires some of the three histone marks associated with transcriptional repression. Histone methyl transferase enzyme (HMT) EZH2 adds the H3K27me3 mark, HMT G9a adds the H3K9me2 mark, and finally, HMT Suv39H1 adds the H3K9me3 mark. In the image, the first five CpG sites (1–5) are represented as methylated CpG sites (either by a DNA methyl transferase 3 or 1 (DNMT 3/1), and these sites are represented as closed circles. The NF-kB binding sites are occupied by the homodimer p50/p50. These homodimers, through the CBF-1 protein, recruit the histone deacetylases (HDAC1 and HDAC2). Additionally, the Sp1 binding sites also recruit HDACs. Finally, it is important to note that the U3 region of the 5′ LTR contains two hormone response elements: the glucocorticoid response element (GRE) and the triiodothyronine response element (T3RE). In the case of T3RE, the interaction of T3Rɑ with T3RE in the absence of ligand allows to recruit HDACs. (**B**) Active State: The CpG sites in the U3 region of the 5′ LTR are unmethylated (these sites are represented as open circles) and are accessible. This allows the binding of transcription factors to the U3 region. The binding of specific transcription factors is dependent on cell type and activation state. In the absence of Tat, but in the presence of activating transcription factors in the U3 region (e.g., the p50/p65 heterodimer at the NF-Kb binding site and Sp1 at the Sp1 binding site) it only enables RNA polymerase II, to generate short transcripts. RNA begins to be transcribed in the R region, and a secondary structure called TAR-RNA is formed. For the generation of long transcripts, the production of the viral protein Tat is necessary, which is post-translationally modified by the enzymes histone acetyl transferases (HATs), and that acetylated Tat interacts first with the TAR RNA, and that later Tat interacts with the RNA pol II. The viral protein Tat is capable of recruiting histones acetyl transferases (such as p300-CBP and hGCN5), and Tat is acetylated at lysine 28 (K28Ac) and at lysine 50 (K50Ac) by the enzyme p300-CBP. Finally, Tat is acetylated at lysine 51 (K51Ac) by the enzyme hGCN5. Once Tat has acquired post-translational modifications, it is recruited to the TAR-RNA region. The K28Ac-mark Tat protein allows it to recruit the p-TEFb complex (consisting of cyclin T1 *CycT1* and *CDK9* kinase). Later, CDK9 kinase phosphorylates Serine 2 residues within the CTD region of RNA pol II. The K50Ac-mark Tat protein allows it to release P-TEFb and recruit the PBAF complex (formed by BAF180/BAF200) to TAR RNA. For RNA pol II to generate long transcripts, the displacement of nuc 1 is necessary. For this, nuc 1 is displaced from the R region to the U5 region of the 5′ LTR. The displacement of nuc 1 is affected by the PBAF complex (BAF 180/BAF 200), and the acetylation of Tat at lysine 51 (K51Ac) allows the recruitment of Tat to RNA pol II (elongation phase). Therefore, the displacement of nuc 1 essentially facilitates the process of elongation of transcription. Histone H3 from nuc 1 acquires some of the marks associated with transcriptional activation. Histone H3 acquires marks given by histone methyl transferases (HMTs). The SET1 enzyme is responsible for adding the H3K4me3 mark, the SETD2 enzyme is responsible for adding the H3K36me3 mark (the SETD2 enzyme is recruited by recognizing the phosphorylated Serine 2 residues in the CTD region of RNApol II). Additionally, Histone H3 from nuc 1 also acquires the marks given by histones acetyl transferases (HATs). HAT p300 is the enzyme responsible for adding the H3K4Ac, H3K9Ac, and H3K27Ac marks to H3. The transcription factors NF-kB and Sp1 recruit to the enzymes histone acetyl transferases (HAT p300). Finally, the hormonal signaling given by T3 and glucocorticoids via response elements in the 5′ LTR helps in the activation process of the viral promoter via the recruitment of the HAT p300 enzyme. (**C**) Epigenomic editing using CRISPR/dCas9-TET/ROS1: CRISPR/dCas9 technology allows the editing of methylated CpG sites in the 5′ LTR via the recruitment of TET or ROS1 enzymes. The 5′ LTR is recognized by specific RNA guides (gRNA LTR), and this system, having a dCas9, lacks the ability to cut DNA. In this Figure, if the 5′ LTR is in a latent state (section A), the promoter can be edited (Section C) and we can move to a 5′ LTR with an active state (section B).

In this review, we outlined a wide range of mechanisms involved in transcriptional regulation and thus HIV-1 latency (Figure 1): 

1. The retrovirus transcription process is regulated by the interactions between numerous different types of viral proteins and cellular transcription factors with binding sites located in the 5′ LTR. The promoter sequence and most of enhancer elements encompass the U3 region. R or U5 regions include additional regulatory elements, which usually increase the strength of the promoter-enhancer region in the U3 and provide a mechanism to broaden the viral response to stimulating factors or regulate transcription in cell-type-dependent manner. 

2. LTRs play a significant role in tissue-specific expression of the proviral genome. Highlighted is the extraordinary capacity of *lentiviruses* to adjust their transcriptional mechanisms to each cell type of the immune system as well as to microglial cells to maximize their replication or adopt a state of proviral latency. The mechanisms underlying HIV-1 latency might differ among different cell lines, as well as latently infected cells that constitute the latent reservoir in HIV-infected individuals with suppressed viral expression due to HAART. It has been proposed that HIV-1 latency may be due to the lack of activation-dependent host cell factors in resting cells, as well as decreased Tat level and microRNAs remodeling LTR promoter-associated chromatin. 

3. Tat is critical for viral infectivity and pathogenesis. Tat expression may play a key role in the establishment and control of a viral reservoir in latently infected resting CD4+ T cells, as well as in cells from the CNS. The modulation of transactivation provoked by Tat sequence variability may have implications on HIV-1 latency and the reactivation phase. However, it remains to be elucidated how CTL escape mutations in Tat sequence may affect HIV-1 latency kinetics at both establishment and reversal phases. 

4. Steroid/thyroid nuclear receptors modulate HIV-1 transcription by distinct molecular mechanisms, depending on the target cell type. Although glucocorticoids can mediate either activation or repression of HIV-1 genes expression, administration or excess secretion of the glucocorticoids is associated with increased HIV-1 replication and accelerated progression towards AIDS.

5. The variability within LTR sequence has a key role in HIV-1 replication and modulation of viral latency, and the reorganization of HIV-1 LTR may influence the transition from a nonprogressive to rapid-progressive stage of infection. Transcription factor binding sites may evolve within LTR to cause differential expression of the virus in particular tissues. Since HIV-1 LTR evolution occurs in a compartmentalized manner, genetically distinct tissue-derived LTRs can be found using phylogenetic analysis. Even subtle alterations in the enhancer-promoter region can play role in attenuating HIV-1 replication during the nonprogressive phase. Moreover, they may contribute to transcriptional silencing in latently infected cells that constitute the viral reservoirs. Particular features within the LTR of non-T cell reservoirs should be considered in clinical approaches intended to kill HIV-1 latently infected cells in patients undergoing HAART.

6. Latency and reactivation of the HIV-1 proviral DNA is influenced by epigenetic mechanisms. A better understanding of the epigenetic characteristics underlying HIV-1 latency establishment (i.e., interrelationships amongst HDACi, DNMTs, and HMTs, chromatin modifiers, and the biochemical mechanisms involved in the repression of the HIV-1 promoter) will aid in the development of more effective latency reversal agents (LRAs) targeting the HIV-1 reservoir. Since DNA methylation could be a late event that strengthens provirus silencing, rather than initiating it, infection time and source cell type could be very important factors in methylation analyses.

7. The lack of selectivity of LRAs is a main limitation for the use of this therapeutic approach in HIV-1-infected patients. Alternatively, the higher specificity and efficiency of CRISPR/Cas9 have led to its widespread application, including in anti-HIV-1 strategies. However, the edition by CRISPR/Cas9 system may cause off-target mutations, and for that reason, the use of CRISPR/dCas9 might be safer due to the absence of DNA damage to the host cell.

## Figures and Tables

**Table 1:** Key transcription factors involved in regulation of human immunodeficiency virus type 1 (HIV-1) transcription in different cell types.

Transcription Factor	Cell Type
**NF-κB**	**T cells** *, monocytes, macrophages, iDC, microglial cells
**NF-AT**	**T cells** *
**Sp1**	**microglial cells** *, **T cells** *, monocytes, macrophages, iDC
**Sp3**	**microglial cells** *, monocytes, macrophages
**AP-1**	**microglial cells** *, monocytes, T cells
**COUP-TF**	**microglial cells** *, T cells
**Ets-1**	**T cells** *
**USF**	**monocytes** *, **macrophages** *, **iDC** *, T cells, microglial cells
**C/EBP (NF-IL-6)**	**monocytes** *, **macrophages** *, **iDC** *, T cells, microglial cells
**CREB/ATF**	**T cells** *, microglial cells, monocytes, macrophages

* transcription factors required for transcriptional activation in cell-type-specific expression of HIV-1; NF-κB, nuclear factor kappa-light-chain-enhancer of activated B cells; NF-AT, nuclear factor of activated T-cells; Sp1, 3, *specific protein* 1, 3; AP-1, activator protein *1*; *COUP-TF,* chicken ovalbumin upstream promoter transcription factor; Ets-1, E26 transformation-specific (ETS) transcription factor; USF, *upstream* stimulatory *factor*; C/EBP, CCAAT/enhancer-binding protein; NF-IL-6, transcription factor nuclear factor interleukin 6; CREB/ATF, cAMP response element-binding protein/activating transcription factor.

**Table 2:** Regulation of HIV-1 gene transcription in primary immune cells and transformed cell lines.

Transcription Factor	Cell Type	Primary Cells	Transformed Cell Line
**NF-κB**	T cells	• activates transcription in dopamine-stimulated PBMCs [11] • activates transcription in CD4+ T cells by direct occupancy of enhancer by NF-κB p50/p65 [12] • activates transactivation in TNF-, IL-1-, and IL-7-stimulated TEC co-cultured with thymocytes [13]	• activates transcription in dopamine-stimulated lymphoid Jurkat T cell line [11] • activates transcription in Jurkat T cell line that stably expresses the Tat [14] • activates transcription in latently HIV-1-infected established T lymphoid cell line J1.1 promoted by MRPs [15]
	monocytes/macrophages	• activates transcription in macrophages by direct occupancy of enhancer by NF-κB p50/p65 [16] • involved in efficient activation of viral transcription in monocytes isolated from PBMC [17]	• activates HIV gene transcription in monocytic cell line U937 and promonocytic cell U1 by direct occupancy of enhancer by NF-κB p50/p65 [18,19]
	microglial cells	nd	• activates transcription in human microglial MC-3 cell line and embryonic microglial cell line upon stimulation with IFNγ, IL1β, and TNFα [20,21,22]
**NF-AT**	T cells	• enhances activation of transcription in CD4+ T cells [12] • NF-AT1,2 enhances activation of transcription in PMA/ionomycin stimulated CD4+ T cells [23] • NFAT1, 2 positive effect on transcription in PMA-, PHA-, bpV-stimulated PBMC [24]	• efficient binding to the HIV-1 LTR enhancer in Jurkat-derived CD4+ T cells isoform CD45(−), stimulated with PMA/PHA/α-CD3 [25] • represses Tat-mediated transactivation in PMA/ionomycin-stimulated Jurkat T-cells [26] • NFAT1, 2 enhances transcription in Jurkat T cells stimulated with PMA, PHA and bpV [24]
**Sp1, 3**	microglial cells	nd	• Sp1 interaction with COUP-TF leads to activation of HIV gene transcription in microglial cell line [18] • binding CTIP-2 to Sp1 represses Tat-mediated transcriptional activation HIV promoter [27] • Sp3 represses Sp1 and COUP-TF-induced activation in human microglial cell line [18]
	T cells	• Sp1 associated with Tat activates transcription in CD4+ T cells and PBMCs [12,28]	• Tat-induced Sp1 activates promoter in MT-2 cell line and Jurkat T cells [28] • Sp1 assembly pre-initiation complex at the LTR TATA box and cooperatively interacts with NF-κB to activate transcription in Jurkat T cells stimulated with PMA [29]
	monocytes/macrophages	• Sp1-to-Sp3 ratio increases during monocyte lineage differentiation, resulting in increased HIV-1 transcription [30]	• Sp1 activates LTR-driven transcription in U1 monocytic cells [31] • Sp1 has moderate impact on transcription activation in human monocytic line U-937 [32]
	iDC	• Sp1 activates HIV gene transcription in DC differentiated from monocytes derived from PBMCs [30,33]	nd
**AP-1**	microglial cells	nd	• c-jun and c-fos interact with TRE sequence and enhance HIV-1 gene transcription in glial cells [34]
	monocytes/macrophages	• Vpr-activated AP-1 enhances viral transcription in macrophages differentiated from PBMCs [35]	• Vpr-activated AP-1 enhances viral transcription in U937 cells [35] • Nuclear complex of c-fos and c-jun binds directly to the HIV LTR and enhances NF-κB activity in human monocytic cell lines U1 and U937 [36] • AP-1 activated by Nef stimulates HIV transcription in U1 and U937 cells [37]
	T cells	• enhances HIV-1 gene expression in CBMCs more than in PBMCs [38]	• c-jun and c-fos do not interact with TRE sequence and do not enhance HIV-1 transcription in Jurkat T cells [34,39]
**COUP-TF**	microglial cells	• cooperates with Tat to promote NF-κB- and Sp1-independent transactivation HIV-1 transcription in human fetal microglial cells [40]	• cooperates with Tat and promotes NF-κB and Sp1-independent activation HIV-1 transcription in microglial cell line [40] • COUP-TF Sp1 interaction stimulates HIV transcription in microglial cell line [18] • COUP-TF, Sp1, and CTIP2 cooperation suppresses HIV transcription initiation in microglial cells [41]
	T cells	nd	• COUP-TF interaction with Sp1 synergistically stimulates viral transcription in Jurkat T cells in response to cAMP and dopamine [42]
**Ets**	T cells	• Ets in cooperation with NF-kB/NFAT activates HIV-1 enhancer in human peripheral blood T cells [43]	• Ets in cooperation with USF-1 enhances transcriptional activity of HIV-1 LTR in Jurkat T cells [44]
**C/EBP** **(NF-IL-6)**	monocytes/macrophages	• regulates HIV transcription by recruiting HATs to the LTR in primary macrophages [45]	• recruits HATs to LTR and mediates initiation of transcription in promonocytic U937 cells [46]
	T cells	nd	• is not required in HIV transcription in Jurkat CD4+ T cell line [45] • cooperates with CREB and mediates prostaglandin E2-induced stimulation of LTR-driven transcription Jurkat E6.1 [47]
	microglial cells	nd	• in presence of IL-1, IL-6, and TNF- α, activates LTR-driven transcription versus C/EBPγ that acts as inhibitor [48]
**CREB**	T cells	• phospho-CREB recruits CBP and basal transcription factors, which increases promoter activation in primary lymphocytes [49]	• phospho-CREB recruits CBP and basal transcription factors, which increases promoter activation in MT-4 human T cell line [50] • mediates cAMP and dopamine-induced transcriptional stimulation through indirect interactions with LTR in Jurkat T cells [42] • cooperates with COUP-TF in the presence of forskolin, cAMP, and dopamine to activate HIV-1 gene transcription in Jurkat T cells [42]
	monocytes/macrophages	nd	• CREB homodimers bind to their DNA site, interact with C/EBPs, and lead to increase HIV promoter activation in U-937 and THP-1 human monocytic cell lines; sequence variations at the CREB site affect LTR activity [51]

nd, not determined; TEC, human thymic epithelial cell; MRPs, proinflammatory myeloid-related proteins; PBMC, peripheral blood mononuclear cell; NF-AT, nuclear factor of activated T cells; PMA, Phorbol 12-myristate 13-acetate; NF-κB, nuclear factor-kappa B; PHA, phytohemagglutinin; bpV, bis-peroxovana-dium a protein tyrosine phosphatases (PTP) inhibitor; COUP-TF, chicken ovalbumin upstream promoter transcription factor; MT-2, cell line derived from normal human cord leukocytes cocultivated with leukemic cells from an adult T cell leukemia (ATL) patient; CBMCs, umbilical cord blood mononuclear cells; CTIP2, Chicken ovalbumin upstream promoter transcription factor interacting protein 2; cAMP, cyclic AMP, adenosine 3’,5’-cyclic monophosphate; HATs, histone acetylotransferase; Ets, erythroblast transformation specific transcription factor; Sp1, transcription factor specificity protein 1; AP-1, activator protein; C/EBP, CCAAT/enhancer-binding protein; NF-IL-6, transcription factor nuclear factor interleukin 6; CREB, cAMP response element-binding protein; CBP, CREB binding protein.
